# Prevalence and determinants of school bullying in Qatar: a cross-sectional study

**DOI:** 10.1186/s12887-023-04227-3

**Published:** 2023-08-16

**Authors:** Madeeha Kamal, Samer Ali, Kholoud Mohamed, Aamir Kareem, Suzan M. Kirdi, Mai Hani, Manasik Hassan, Schahla Al-Shibli, Prem Chandra

**Affiliations:** 1 Sidra Medicine, Doha, Qatar; 2 Hamad Medical Corporation, Doha, Qatar; 3 University of Toronto, Toronto, ON Canada

**Keywords:** Bullying, Schools, Qatar

## Abstract

**Background:**

School bullying is a wide-spread phenomenon that manifests in various forms. It has both short-term and long-term devastating consequences on physical, mental and social wellbeing. The Middle East and North Africa (MENA) region, including Qatar, has a relatively high prevalence of school bullying. This research aims at identifying the prevalence of bullying, particularly unsafe environments were bullying takes place, and its attributes at schools in Qatar.

**Methods:**

In a cross-sectional study, 980 students from 10 schools in Qatar completed an anonymous self-completion standardized questionnaire to assess the different aspects of bullying from school students’ point of view.

**Results:**

The prevalence of bullying victimization and perpetration was found to be 41.0% and 31.7% among school students in Qatar, respectively. Classroom (67.5%) and hallways (64.8%) were the most frequently indicated environments of bullying whereas library was the least indicated one (28.3%). Verbal bullying was the most used type of bullying by students. Overall, students in Qatar believe that bullying is considerably a significant issue at their schools, yet schools are safe place for them to be in. Gender, age, ethnicity, school grade and years living in Qatar showed significant differences among the students.

**Conclusion:**

School bullying is a serious, yet a manageable global problem. Our findings re-demonstrated the alarming high prevalence of school bullying in Qatar, highlighted student related and school related factors which have implications for future multidimensional action and research and recommended measures to foster safety at school.

**Supplementary Information:**

The online version contains supplementary material available at 10.1186/s12887-023-04227-3.

## Introduction

Bullying is essentially a form of Adverse Childhood Experiences [1]. It is characterized by a repeated aggression or intentional harming over time in which one or group of persons have a greater power over one or a group who are helpless in defending themselves. Bullying, or so called “traditional bullying”, can take many forms including verbal (e.g., teasing, name-calling), physical (e.g., hitting, tripping), social (e.g., spreading rumors, leaving someone out on purpose) and sexual (e.g., unwanted touching, gestures) [2]. In the last decade, bullying has even evolved to take a new form on the online network as what is known as cyberbullying where at least one in ten children has been affected [3]. Unfortunately, bullying has become a wide-spread phenomenon as one in three students globally has been bullied at least once [3]. Moreover, aside from the instant direct injury that can result from physical bullying, the effect of the different types of bullying has a dramatic impact on the child overall health and wellbeing. An overwhelming body of research has found that bullying victims are at a higher risk of developing depressive symptoms, somatization, self-harm behavior, body pains or headaches, of living alone and of abusing drugs and tobacco to name a few [3–6]. Thus, the extent of the problem is of huge magnitude and its consequences are of deep concern.

Globally, the prevalence of bullying varies widely. On one hand, Europe and North America have witnessed a remarkable decline of bullying overtime which reflects the overall global trend, yet, an enormous amount of work and legislation had led to this positive shift [2]. On the other hand, the Middle East and North Africa (MENA) region comes in second and third places as highest reported prevalence of bullying worldwide where race and ethnicity are the most common drivers of bullying in both genders, and it is on the rise [2]. In the gulf region, bullying is highly prevalent as in Oman (47.4%), Kuwait (28.3%) and United Arab Emirates (22.8%) as reported by the Global School-based Student Health Survey (GSHS) of the World Health Organization [7]. Locally, the GSHS reported that 42.2% of students in Qatar had been bullied on one or more days during the past month. Nonetheless, there is a significant lack of an updated estimate of the school bullying in Qatar and other GCC countries, which have similar sociodemographic features and are expected to have similar pattern of bullying.

### Aim of this study

The present study aims at filling the aforementioned gap by identifying the prevalence of bullying and its attributes at schools in Qatar, in terms of student’s sociodemographic characteristics, their status of bullying involvement, the bullying types indicated, the specific school environments where bullying might take place, the students’ feeling of safety at school and the students’ perception of bullying. Ultimately, our goal is to enhance the safety of students at school.

## Methods

### Study design and sampling procedure

This is a cross-sectional descriptive study that covered ten public and private schools in Qatar distributed in the capital of Qatar, Doha, at its suburbs. After obtaining ethical approval from Hamad Medical Corporation Ethics Committee and the Supreme Education Council Research Office, all of schools in Qatar were emailed with study aims and design and 10 school principals’ showed willingness to participate in the study. In each school, parents or legal guardians were contacted via SMS to obtain consent for their children to participate in the study. Students whom parents or legal guardians did not consented to participate were excluded from the study. Participants grades ranged from the fourth to the twelfth grade. Students were grouped into 3 school grade level groups: Elementary [1–6], preparatory [7–9] and secondary or high schools [10–12], which is in accordance to how students proceed through the educational system in Qatar, that is also similar for most countries in the Middle East. It is important to highlight here that students’ grades differ from students’ age. In Qatar, students are allowed to repeat the academic year twice if they fail to pass the minimum requirement which allows some students to be elder compared to their peers in each class.

In each school, the research team approached the given school class, which on average includes 27 students, and administered the questionnaire to the students. No time limit was given to the students to fill out the questionnaire and each student given clarification if any needed. However, in elementary school class, the questionnaire was read aloud in both languages Arabic and English by the class teacher and further explanation was given by research team members when needed. Students were encouraged to fill out the whole survey and given the freedom to stop at any time.

### The questionnaire

An anonymous self-completion standardized paper-based questionnaire was utilized to assess different aspects of bullying at schools. The questionnaire was developed by experts in the field of pediatrics mental health benefiting from Olweus Bully/Victim questionnaire and other multiple similar tools of surveying students on their involvement in bullying at schools from the literature [8–11]. The rationale behind developing a questionnaire derived from multiple sources was to ensure maximum appropriateness of the questions to be culturally sensitive to Qatari society. Containing 80 items, the questionnaire consisted of multiple choice and Likert scale-based questions given in both languages Arabic and English based on the student’s preference. Apart from the demographics, the questions assessed different aspects of bullying including the students’ involvement status (victim, perpetrator or observer) in the past four weeks, bullying types, the specific environment where bullying might take place, the students’ feeling of safety at school and students’ perception of bullying’s significance. The rationale behind choosing these categories of bullying involvement status based on the previous research classification that highlighted the importance of study each sub-group and their risk factors [12–15]. The aforementioned aspects were addressed via multiple choice questions. The rest of the 80 questions were outside the scope of this paper.

The concept of bullying can be perceived differently among different ages. Additionally, bullying is not well-addressed in the Qatari society especially with its given jargon Arabic translation and its various definitions that can be misleading. Accordingly, a brief 2-pages long consisted of bullying-related definitions and its types with examples of each type were provided ahead of the questions to ensure a common understanding of bullying among the students answering the questionnaire using an easy student-level language and utilizing Olweus definition as a common reference [8]. Kindly refer to the attached supplementary materials for more details. The questionnaire was translated into Arabic dialect as used in Qatar and back translated into English by 3 different independent physicians in the field of pediatrics to ensure the meaning remained unchanged. Taking in consideration safety, conditionality, comfortableness and clear understanding of the questions, the research team accompanied classroom teachers to administer the questionnaires.

### Sample size determination

As reported in the recent literature on the Gulf region from the WHO GSHS [7], the prevalence of bullying in the region are varied and ranged between 30 and 40%. Hence, the sample size was computed using expected prevalence of bullying is 35% and precision of estimate (margin of error) is 3% and level of confidence (1-α) is 95%, the sample size required for this study would be 971 participants.

The following statistical formula [16] was used in computing the required sample size.

1 $$n\, = \,\left[ {{Z^2}_{1 - \alpha /2}P\left( {1 - P} \right)\,/\,{d^2}} \right]$$

Where n = sample size, Z = Z statistic for a level of confidence (for the level of confidence of 95%, which is conventional, Z value is 1.96), P = expected prevalence or proportion and d = precision (in proportion of one; if 5%, d = 0.05).

### Statistical data analysis

Descriptive statistics, namely frequencies and percentages, were used to report population characteristics, bullying involvement status, bullying types, frequency of bullying occurrence in different environments, bully characteristics, and student’s perception of bullying. Associations between two or more qualitative data variables were assessed using Chi-Square (χ2) test or Fisher’s exact test as appropriate. Univariate and multivariate logistic regression analysis was used to investigate sociodemographic determinants of bullying involvement status and student’s perception of bullying, with adjustment to control for potential predictors and confounding factors namely gender, ethnicity, age group, school grade and years lived in Qatar, predictor variables were included considering both statistical and clinical significance. All P-values presented were two-tailed, and P-values < 0.05 was considered as statistically significant. All Statistical analyses were done using statistical packages SPSS version 27.0 (Armonk, NY: IBM Corp) and Epi-info (Centers for Disease Control and Prevention, Atlanta, GA) software.

### Reliability

Strong internal reliability for each of the 3 domains (victim, perpetrator and observer) was confirmed by computing Cronbach alpha statistic for each domain. Cronbach alpha values were 0.866 (95% CI 0.847, 0.884), 0.846 (95% CI 0.822, 0.868) and 0.812 (95% CI 0.787, 0.835) respectively thereby demonstrating high internal reliability.

## Results

### Participant’s characteristics

Data showed that 980 students from 5 governmental and 5 private schools had successfully completed the questionnaire. As detailed in Table 1, males comprised 60.7% of the whole sample. Ethnicity varied among the students as 58.6% of the students were Arabs, 14.6% were Asian/Caucasians and 26.8% were labeled “Other” for their ethnicity. Most students aged between 11 and 15 years and the majority of students were students in the preparatory level (60.1%). In addition, the majority of students had lived more than 10 years in Qatar (60.2%).

**Table 1:** Population Characteristics

		*N* = 980*	%
Gender			
	Males	577	60.7
	Females	374	39.3
Ethnicity			
	Arab	555	58.6
	Asian/Caucasian	138	14.6
	Other	254	26.8
Age Group			
	8–10 Years	43	4.4
	11–15 Years	765	78.4
	16–18 Years	168	17.2
School Grade			
	Elementary	189	19.4
	Preparatory	586	60.1
	Secondary	200	20.5
Years lived in Qatar			
	Less than 5 years	119	12.6
	5–10 Years	257	27.2
	More than 10 years	568	60.2

*The number/frequencies for some variables may not add-up to a total n = 980 due to missing observations (unavailability of the information). All percentage (%) values were computed using non-missing data values. This applies to all below tables.

### Bullying involvement status

Among the 980 students, 402 (41.0%) students were victimized and 275 (31.7%) were perpetrators of bullying. Out of these students, 156 (18.7%) students were victims as well as perpetrators. Lastly, 579 (76.3%) had at least observed bullying at school in the past four weeks.

### Bullying types

**Fig. 1:**
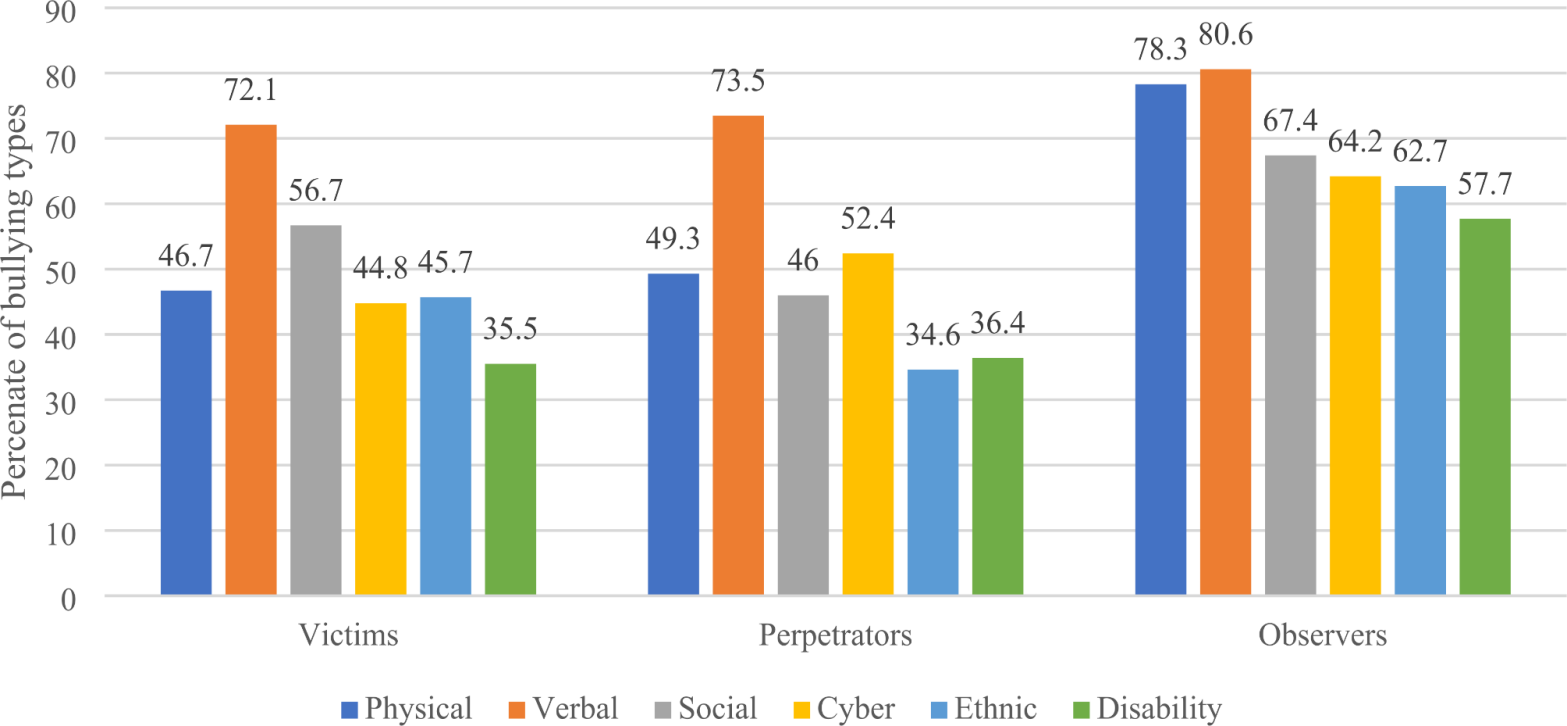
Prevalence of Bullying Types

The prevalence of bullying types that is shown in Fig. (1) indicates that the most common type of bullying is verbal among victims (72.1%), perpetrators (73.5%) and observers (80.6%). Contrarily, disability bullying was the least common among victims (35.5%) and observers (57.7%) but ethnic bullying was the least common among perpetrators (34.6%).

Studying each type of bullying against the students sociodemographic’ showed significant differences as Tables 2, 3 and 4 illustrate.

For students who were victimized, males were significantly more likely to be subjected to verbal, cyber, ethnic and disability bullying compered to females (P < 0.05). In addition, preparatory school grade students were more likely to be exposed to all types of bullying compared to other school grades (P < 0.05).

For bullying perpetrators, males were more likely to practice all types of bullying compared to females. Arab student practiced physical, ethnic and disability bullying significantly more than other ethnicities. In addition, preparatory school grades students used verbal, physical, social and cyber bullying types. However, elementary students were more likely to practice other forms of bullying namely ethnic and disability. Perpetrators lived in Qatar more than 10 years were more likely to use all types of bullying compared to other student except social bullying (P < 0.05).

For students who observed bullying in school, males observed more verbal and social bullying compared to females (P < 0.05). Students aged 16 to 18 years were more likely to observe cyber bullying compared to other age groups. In addition, secondary grade student observed more verbal, social and cyber bullying at their schools compared to other school grades (P < 0.05). Lastly, students who lived in Qatar for 5 to 10 years were more likely to observe ethnic bullying (P < 0.05).

**Table 2:** Sociodemographic Correlates of Bullying Types Among Victims: Chi-Square Analysis

	Physical N (%)	Verbal N (%)	Social N (%)	Cyber N (%)	Ethnic N (%)	Disability N (%)
Gender						
Males	307 (62.9)	226 (46.2)	217 (44.7)	162 (33.1)	176 (36.1)	122 (25.4)
Females	190 (57.1)	81 (24.3)	167 (49.3)	84 (24.7)	92 (26.9)	64 (19.1)
**χ**^**2**^**(P-value)**	2.84 (0.092)	**40.9 (< 0.001)**	1.71 (0.919)	**6.82 (0.009)**	**7.84 (0.005)**	**4.4 (0.037)**
Ethnicity						
Arab	187 (38)	291 (59.8)	234 (47.4)	152 (30.8)	151 (30.1)	120 (24.3)
Asian/Caucasian	45 (37.5)	71 (60.2)	54 (46.2)	27 (23.1)	40 (33.9)	17 (14.7)
Other	64 (31.4)	121 (58.5)	91 (43.8)	61 (28.8)	70 (33.2)	48 (23.2)
**χ**^**2**^**(P-value)**	2.84 (0.24)	0.13 (0.94)	0.77 (0.68)	2.76 (0.25)	1.02 (0.60)	5.07 (0.08)
Age Group						
8–10 Years	20 (60.6)	16 (48.5)	15 (45.5)	12 (36.4)	14 (46.7)	10 (35.7)
11–15 Years	403 (61.2)	243 (36.9)	317 (47.9)	196 (29.5)	207 (31)	154 (23.4)
16–18 Years	80 (54.1)	51 (22.6)	57 (37.7)	42 (27.8)	50 (32.7)	24 (16.2)
**χ**^**2**^**(P-value)**	2.61 (0.271)	2.63 (0.27)	5.09 (0.08)	0.95 (0.62)	3.26 (0.19)	6.58 (0.073)
School Grade						
Elementary	111 (49.4)	93 (59.2)	95 (58.3)	69 (42.3)	67 (42.4)	64 (40.5)
Preparatory	297 (59.6)	157 (31.3%)	227 (45.5)	138 (27.5)	148 (29.2)	107 (21.4)
Secondary	93 (59.9)	59 (32.2%)	65 (45.7)	43 (23.6)	56 (31)	18 (10.2)
**χ**^**2**^**(P-value)**	**10.7 (0.005)**	**42.1 (< 0.001)**	**17.7 (< 0.001)**	**16.9 (< 0.001)**	**8.4 (0.02)**	**44.96 (< 0.001)**
Years lived in Qatar						
Less than 5 years	58 (53.7)	30 (28)	46 (42.2)	32 (29.4)	28 (26.2)	20 (18.5)
5–10 Years	134 (64.7)	85 (39.7	107 (50)	63 (29)	76 (34.9)	45 (21.4)
More than 10 years	290 (58.5)	180 (36.4)	219 (44.2)	145 (29.3)	153 (38.8)	117 (23.8)
**χ**^**2**^**(P-value)**	4.08 (0.13)	4.25 (0.12)	2.54 (0.28)	0.006 (0.997)	2.66 (0.27)	1.58 (0.45)

**Table 3:** Sociodemographic Correlates of Bullying Types Among Perpetrators: Chi-Square Analysis

	Physical N (%)	Verbal N (%)	Social N (%)	Cyber N (%)	Ethnic N (%)	Disability N (%)
Gender						
Males	133 (31.4)	188 (45.3)	118 (28.4)	140 (32.9)	104 (24.5)	102 (24.5)
Females	33 (10.3)	86 (27)	48 (14.9)	34 (10.7)	17 (5.2)	14 (4.3)
**χ**^**2**^**(P-value)**	**36.6 (< 0.001)**	**25.94 (< 0.001)**	**19.04 (< 0.001)**	**49.56 (< 0.001)**	**50.58 (< 0.001)**	**55.75 (< 0.001)**
Ethnicity						
Arab	114 (26.1)	168 (39.3)	105 (24)	111 (25.1)	85 (19.1)	78 (17.7)
Asian/Caucasian	18 (15.3)	32 (27.1)	13 (11)	19 (16.1)	9 (7.6)	10 (8.5)
Other	34 (17.6)	69 (36.3)	44 (23.3)	39 (20.7)	24 (12.8)	24 (12.9)
**χ**^**2**^**(P-value)**	**9.47 (0.009)**	5.88 (0.053)	9.57 (0.008)	4.82 (0.09)	**10.99 (0.004)**	**7.0 (0.03)**
Age Group						
8–10 Years	9 (36)	10 (38.5)	9 (32.1)	8 (30.8)	4 (20.8)	5 (20.8)
11–15 Years	126 (21.2)	218 (57.5)	124 (21.2)	132 (22.3)	93 (15.5)	93 (15.8)
16–18 Years	33 (23.1)	48 (33.3)	33 (22.8)	35 (24)	23 (16)	18 (12.6)
**χ**^**2**^**(P-value)**	3.16 (0.21)	0.91 (0.636)	1.96 (0.373)	1.13 (0.57)	0.50 (0.778)	1.47 (0.48)
School Grade						
Elementary	60 (45.8)	73 (52.9)	59 (42.1)	62 (43.7)	60 (42.9)	55 (40.4)
Preparatory	77 (16.9)	146 (33.4)	75 (17)	77 (17.3)	40 (8.8)	48 (10.8)
Secondary	33 (18.8)	57 (32.4)	33 (18.6)	37 (20.9)	22 (12.7)	14 (8)
**χ**^**2**^**(P-value)**	**50.6 (< 0.001)**	**19.02 (< 0.001)**	**40.78 (< 0.001)**	**42.60 (< 0.001)**	**94.4 (< 0.001)**	**79.8 (< 0.001)**
Years lived in Qatar						
Less than 5 years	12 (12.5)	23 (24.7)	18 (18.8)	12 (12.6)	9 (9.4)	10 (10.6)
5–10 Years	41 (20.4)	72 (36.5)	39 (19.6)	32 (18.6)	22 (11.2)	21 (10.8)
More than 10 years	107 (24.2)	174 (39.6)	102 (23.1)	120 (26.7)	84 (18.6)	81 (18.2)
**χ**^**2**^**(P-value)**	**6.56 (0.038)**	**7.34 (0.026**)	1.54 (0.462)	**11.4 (0.003)**	**8.96 (0.011)**	**7.48 (0.024)**

**Table 4:** Sociodemographic Correlates of Bullying Types Among Observers: Chi-Square Analysis

	Physical N (%)	Verbal N (%)	Social N (%)	Cyber N (%)	Ethnic N (%)	Disability N (%)
Gender						
Males	282 (70.5)	279 (69.6)	226 (56.8)	214 (53.6)	202 (50.9)	163 (41.3)
Females	230 (69.9)	257 (78.6)	216 (66.3)	171 (52.6)	174 (52.7)	126 (39)
**χ**^**2**^**(P-value)**	0.30 (0.86)	**7.54 (0.006)**	**6.76 (0.009)**	0.08 (0.79)	0.25 (0.62)	0.38 (0.54)
Ethnicity						
Arab	299 (70.2)	317 (74.9)	260 (61.9)	228 (54)	222 (53.0)	176 (42)
Asian/Caucasian	80 (70.8)	86 (75.4)	68 (60.2)	60 (52.6)	56 (48.3)	42 (37.5)
Other	134 (69.2)	134 (68.7)	114 (58.8)	96 (49.7)	97 (49.7)	70 (36.6)
**χ**^**2**^**(P-value)**	0.069 (0.97)	2.94 (0.23)	0.57 (0.75)	0.98 (0.61)	1.09 (0.58)	1.89 (0.39)
Age Group						
8–10 Years	21 (75)	16 (59.3)	14 (51.9)	16 (57.1)	14 (51.9)	13 (46.4)
11–15 Years	404 (70.5)	442 (73.0	342 (59.5)	283 (49.4)	294 (51.1)	224 (40)
16–18 Years	97 (66.4)	106 (76.3)	90 (65.2)	89 (63.1)	73 (51.8)	54 (37.5)
**χ**^**2**^**(P-value)**	1.28 (0.53)	3.34 (0.188)	2.36 (0.307)	**8.83 (0.012)**	0.02 (0.99)	0.84 (0.66)
School Grade						
Elementary	81 (65.9)	82 (59.9)	63 (47)	64 (47.4)	69 (51.9)	68 (50.7)
Preparatory	316 (71.2)	329 (75.3)	270 (62.1)	217 (50)	217 (50)	158 (37.5)
Secondary	126 (70)	134 (78.8)	114 (67.1)	108 (62.4)	96 (54.5)	66 (37.3)
**χ**^**2**^**(P-value)**	1.30 (0.52)	**16.17 (< 0.001)**	**13.7 (0.001)**	**9.33 (0.009)**	1.05 (0.59)	**8.06 (0.02)**
Years lived in Qatar						
Less than 5 years	66 (65.3)	72 (71.3)	64 (62.7)	50 (48.5)	46 (44.7)	31 (30.4)
5–10 Years	158 (76.3)	152 (74.1)	119 (59.2)	105 (51)	121 (59.3)	84 (42)
More than 10 years	288 (68.2)	309 (73.4)	251 (59.9)	223 (53.5)	206 (49.2)	169 (40.8)
**χ**^**2**^**(P-value)**	5.61 (0.06)	0.29 (0.867)	0.37 (0.83)	0.94 (0.62)	**7.83 (0.02)**	4.35 (0.11)

### Frequency of bullying in different school environments and activities

**Fig. 2:**
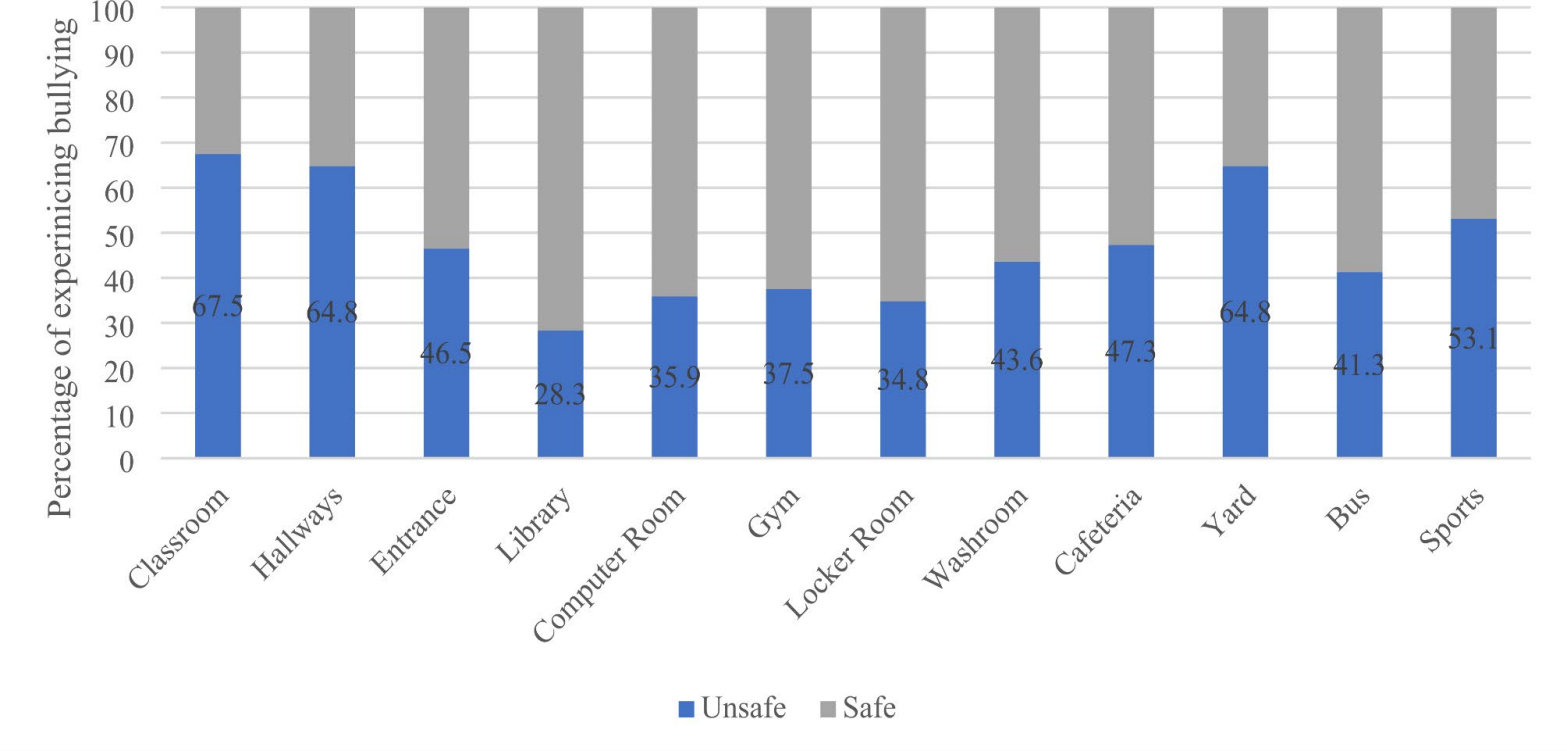
Frequency of Bullying in Different Environments

Among 12 identified different locations and school-based activities, students were asked to report how often did they see or experience bullying in the past 4 weeks on a range of never, once or twice, three or four times and more than four times. If bullying happened in a certain place at least once or twice or more, it was considered to be unsafe. Figure 2 summarized the frequency of bullying occurrence at different school environments. Classroom (67.5%) and hallways (64.8%) were the most frequently indicated environments of bullying whereas library was the least indicated one (28.3%).

### Bullying status determinants (sociodemographic characteristics)

The results of univariate and multivariate logistic regression analysis of sociodemographic determinants of bullying involvement status are presented in Tables 5, 6, 7 and 8. Statistical significance was altered for different variables after adjustment for confounding bias.

Compared to males, females were significantly less likely to be victims of bullying (Adjusted odds ratio (AOR) 0.68, 95% Confidence interval (CI) 0.50, 0.92; P = 0.013), perpetrators (AOR 0.38, 95% CI 0.27, 0.54; P < 0.001) or victims-perpetrators (AOR 0.35, 95% CI 0.22, 0.55; P < 0.001), but were more likely to be observers although statistically was not significant (AOR 1.38, 95% CI 0.94, 2.00; P = 0.097). Compared to Arabs, Asian/Caucasian students were significantly more likely to be victims (AOR 1.56, 95% CI 1.0, 2.45; P = 0.049) or victims-perpetrators (AOR 1.90, 95% CI 1.05, 3.43; P = 0.034). Compared to students aged 8–10, students aged 11–15 and students aged 16–18 were significantly more likely to be observers of bullying with ratios of (AOR 2.39, 95% CI 1.33, 4.30; P = 0.004) and (AOR 1.74, 95% CI 1.03, 2.94; P = 0.038), respectively.

Compared to elementary grades students, students in preparatory and secondary levels were significantly less likely to be victims with ratios of (AOR 0.61, 95% CI 0.40, 0.92; P = 0.019) and (AOR 0.50, 95% CI 0.26, 0.94; P = 0.032), respectively. Secondary school students were significantly less likely to be perpetrators (AOR 0.43, 95% CI 0.21, 0.85; P = 0.016) or victims-perpetrators (AOR 0.23, 95% CI 0.09, 0.58; P = 0.002). Compared to students who lived in Qatar for less than 5 years, students who lived for 5–10 years or for more than 10 years were significantly more likely to be victims with ratios of (AOR 1.94, 95% CI 1.18, 3.20; P = 0.009) and (AOR 1.75, 95% CI 1.10, 2.82; P = 0.023), respectively. In addition, they were significantly more likely to be observers with ratios of (AOR 2.39, 95% CI 1.33, 4.30; P = 0.004) and (AOR 1.74, 95% CI 1.03, 2.94; P = 0.038), respectively. Students who lived in Qatar for more than 10 years were significantly more likely to be perpetrators (AOR 2.85, 95% CI 1.56, 5.22; P = 0.001) or victims-perpetrators (AOR 3.10, 95% CI 1.38, 6.96; P = 0.006).

**Table 5:** Sociodemographic correlates of bullying involvement status (Victims): Univariate and Multivariate Logistic regression analysis

	N (%)	Unadjusted Odds Ratio (95% CI)	P-value	Adjusted Odds Ratio (95% CI)	P-value
Gender					
Males	257 (47.3)	1.0 (reference)		1.0 (reference)	
Females	133 (36.4)	0.638 (0.49, 0.84)	0.001	0.68 (0.50, 0.92)	**0.013**
Ethnicity					
Arab	216 (40.4)	1.0 (reference)		1.0 (reference)	
Asian/Caucasian	64 (50.8)	1.52 (1.03, 2.25)	0.034	1.56 (1.0, 2.45)	**0.049**
Other	109 (44.7)	1.19 (0.88, 1.62)	0.259	1.32 (0.93, 1.86)	0.117
Age Group					
8–10 Years	23 (56.1)	1.0 (reference)		1.0 (reference)	
11–15 Years	332 (45.4)	0.65 (0.35, 1.22)	0.182	1.07 (0.48, 2.32)	0.874
16–18 Years	45 (28.5)	0.312 (0.15, 0.63)	0.001	0.69 (0.26, 1.84)	0.456
School Grade					
Elementary	102 (58.3)	1.0 (reference)		1.0 (reference)	
Preparatory	241 (42.5)	0.53 (0.38, 0.75)	< 0.001	0.61 (0.40, 0.92)	**0.019**
Secondary	58 (30.9)	0.32 (0.21, 0.49)	< 0.001	0.50 (0.26, 0.94)	**0.032**
Years lived in Qatar					
Less than 5 years	38 (32.5)	1.0 (reference)		1.0 (reference)	
5–10 Years	122 (49.6)	2.05 (1.29, 3.24)	0.002	1.94 (1.18, 3.20)	**0.009**
More than 10 years	225 (41.8)	1.49 (0.98, 2.28)	0.063	1.75 (1.1, 2.82)	**0.023**

**Table 6:** Sociodemographic Correlates of Bullying Involvement Status (Perpetrator): Univariate and Multivariate Logistic Regression Analysis

	N (%)	Unadjusted Odds Ratio (95% CI)	P-value	Adjusted Odds Ratio (95% CI)	P-value
Gender					
Males	205 (41.7)	1.0 (reference)		1.0 (reference)	
Females	65 (18.6)	0.319 (2.23, 0.44)	< 0.001	0.38 (0.27, 0.54)	**< 0.001**
Ethnicity					
Arab	167 (33.9)	1.0 (reference)	0.222	1.0 (reference)	
Asian/Caucasian	35 (27.6)	0.74 (0.48, 1.14)	0.177	1.40 (0.85, 2.31)	0.182
Other	64 (28.7)	0.77 (0.56, 1.11)	0.171	1.21 (0.81, 1.79)	0.356
Age Group					
8–10 Years	13 (39.4)	1.0 (reference)	0.576	1.0 (reference)	
11–15 Years	209 (31.0)	0.69 (0.34, 1.41)	0.310	0.75 (0.31, 1.80)	0.516
16–18 Years	51 (32.5)	0.74 (0.24, 1.61)	0.446	1.17 (0.40, 3.43)	0.782
School Grade					
Elementary	67 (44.7)	1.0 (reference)	0.001	1.0 (reference)	
Preparatory	153 (29.4)	0.52 (0.36, 0.75)	< 0.001	0.73 (0.46, 1.14)	0.166
Secondary	52 (26.8)	0.45 (0.29, 0.71)	0.001	0.43 (0.21, 0.85)	**0.016**
Years lived in Qatar					
Less than 5 years	21 (19.1)	1.0 (reference)	< 0.001	1.0 (reference)	
5–10 Years	58 (25.4)	1.45 (0.83, 2.53)	0.198	1.80 (0.95, 3.42)	0.073
More than 10 years	185 (37.0)	2.49 (1.50, 4.14)	< 0.001	2.85 (1.56, 5.22)	**0.001**

**Table 7:** Sociodemographic Correlates of Bullying Involvement Status (Victim-Perpetrator): Univariate and Multivariate Logistic regression Analysis

	N (%)	Unadjusted Odds Ratio (95% CI)	P-value	Adjusted Odds Ratio (95% CI)	P-value
Gender					
Males	120 (25.6)	1.0 (reference)		1.0 (reference)	
Females	31 (9.0)	0.29 (0.19, 0.44)	< 0.001	0.35 (0.22, 0.55)	**< 0.001**
Ethnicity					
Arab	95 (20.0)	1.0 (reference)	0.214	1.0 (reference)	
Asian/Caucasian	24 (20.2)	1.01 (0.61, 1.67)	0.967	1.90 (1.05, 3.43)	**0.034**
Other	32 (14.6)	0.68 (0.44, 1.06)	0.089	1.07 (0.65, 1.76)	0.793
Age Group					
8–10 Years	11 (33.3)	1.0 (reference)	0.026	1.0 (reference)	
11–15 Years	124 (19.1)	0.47 (0.22, 0.99)	0.050	0.47 (1.18, 1.23)	0.126
16–18 Years	20 (13.3)	0.31 (0.13, 0.73)	0.007	0.69 (0.189, 2.52)	0.572
School Grade					
Elementary	46 (31.9)	1.0 (reference)	< 0.001	1.0 (reference)	
Preparatory	91 (18.1)	0.47 (0.31, 0.71)	< 0.001	0.70 (0.42, 1.16)	0.167
Secondary	19 (10.3)	0.244 (0.14, 0.44)	< 0.001	0.23 (0.09, 0.58)	**0.002**
Years lived in Qatar					
Less than 5 years	12 (11.1)	1.0 (reference)	0.021	1.0 (reference)	
5–10 Years	35 (15.8)	1.50 (0.74, 3.01)	0.259	1.83 (0.78, 4.28)	0.166
More than 10 years	103 (21.5)	2.20 (1.16, 4.16)	0.016	3.10 (1.38, 6.96)	**0.006**

**Table 8:** Sociodemographic Correlates of Bullying Involvement (Observer): Univariate and Multivariate Logistic Regression Analysis

	N (%)	Unadjusted Odds Ratio (95% CI)	P-value	Adjusted Odds Ratio (95% CI)	P-value
Gender					
Males	316 (73.3)	1.0 (reference)		1.0 (reference)	
Females	263 (80.2)	1.47 (1.04, 2.08)	0.028	1.38 (0.94, 2.00)	0.097
Ethnicity					
Arab	340 (75.6)	1.0 (reference)	0.127	1.0 (reference)	
Asian/Caucasian	99 (83.2)	1.60 (0.95, 2.71)	0.890	1.50 (0.82, 2.74)	0.184
Other	143 (73.3)	0.90 (0.61, 1.31)	3.091	0.88 (0.57, 1.35)	0.547
Age Group					
8–10 Years	25 (86.2)	1.0 (reference)	0.186	1.0 (reference)	
11–15 Years	466 (76.8)	0.53 (0.18, 1.55)	0.244	2.39 (1.33, 4.30)	**0.004**
16–18 Years	103 (71.5)	0.40 (0.13, 1.23)	0.109	1.74 (1.03, 2.94)	**0.038**
School Grade					
Elementary	97 (74.6)	1.0 (reference)	0.661	1.0 (reference)	
Preparatory	367 (77.4)	1.167 (0.74, 1.83)	0.501	1.42 (0.85, 2.38)	0.181
Secondary	132 (74.6)	0.99 (0.59, 1.68)	0.994	1.38 (0.63, 3.02)	0.427
Years lived in Qatar					
Less than 5 years	69 (67.6)	1.0 (reference)	0.003	1.0 (reference)	
5–10 Years	179 (84.0)	2.51 (1.45, 4.38)	0.001	2.39 (1.33, 4.30)	**0.004**
More than 10 years	336 (75.0)	1.44 (0.90, 2.29)	0.129	1.74 (1.03, 2.94)	**0.038**

### Students’ perception of bullying and its determinants (sociodemographic characteristics)

Student’s perception of bullying problem was assessed through two questions. First question addressed the feeling of safety at school with the statement of “I feel safe at school”. Those who answered “strongly agree” or “kind of agree” were classified as agree/feeling safe, while those who answered “strongly disagree” or “kind of disagree” were classified as disagree/feeling unsafe. Over three-quarters of the students (78.2%) felt safe at school.

Second question addressed the extent to which students believe that bullying is considered as a significant problem at their respective school, with the statement of “how much of a problem do you think bullying is at your school?”. Those who indicated “the biggest problem we have” or “big problem” or “medium problem” were classified as agree/ significant problem whereas those who indicated “small problem or “not a problem at all” were classified as disagree/not a significant problem. The majority of students (76.0%) believed that bullying is a significant problem at school.

Overall, students in Qatar believe that bullying is considerably a significant issue at their schools, yet schools are safe place for them to be in.

The results of univariate and multivariate logistic regression analysis of sociodemographic determinants of students’ perception of bullying are presented in Tables 9 and 10. Statistical significance was altered for different variables after adjustment for confounding bias.

Compared to males, females were significantly more likely to feel safe at their respective schools (AOR 1.54, 95% CI 1.07, 2.22; P = 0.019). Nonetheless, they felt that bullying is a significant problem at their schools (AOR 1.54, 95% CI 1.16, 2.52; P = 0.006). Compared to Arab students, other ethnicities were significantly more likely to feel safe at schools (AOR 1.59, 95% CI 1.03, 2.45; P = 0.035). Compared to students who lived in Qatar for less than 5 years, students who lived for 5–10 year were significantly more likely to be feel that bullying is a significant problem at school (AOR 1.93, 95% CI 1.06, 3.45; P = 0.030).

**Table 9:** Sociodemographic Correlates of Students Feeling Safe at School: Univariate and Multivariate Logistic Regression Analysis

	N (%)	Unadjusted Odds Ratio (95% CI)	P-value	Adjusted Odds Ratio (95% CI)	P-value
Gender					
Males	421 (75.3)			1.0 (reference)	
Females	307 (83.0)	1.58 (1.15, 2.22)	0.006	1.54 (1.07, 2.22)	**0.019**
Ethnicity					
Arab	413 (75.8)	1.0 (reference)	0.043	1.0 (reference)	
Asian/Caucasian	108 (80.0)	1.28 (0.80, 2.03)	0.301	1.15 (0.68, 1.93)	0.606
Other	208 (83.5)	1.62 (1.10, 2.39)	0.015	1.59 (1.03, 2.45)	**0.035**
Age Group					
8–10 Years	30 (69.8)	1.0 (reference)	0.370	1.0 (reference)	
11–15 Years	589 (78.7)	1.60 (0.82, 3.15)	0.169	1.95 (0.79, 4.82)	0.148
16–18 Years	126 (77.3)	1.48 (0.67, 3.11)	0.307	1.30 (0.42, 4.01)	0.647
School Grade					
Elementary	142 (79.3)	1.0 (reference)	0.476	1.0 (reference)	
Preparatory	444 (76.7)	0.86 (0.57, 1.3)	0.460	0.65 (0.387, 1.08)	0.093
Secondary	157 (80.5)	1.08 (0.65, 1.79)	0.775	1.20 (0.55, 2.62)	0.649
Years lived in Qatar					
Less than 5 years	91 (77.1)	1.0 (reference)	0.796	1.0 (reference)	
5–10 Years	202 (79.5)	1.15 (0.68, 1.95)	0.598	1.04 (0.58, 1.77)	0.844
More than 10 years	429 (77.6)	1.03 (0.64, 1.65)	0.914	1.10 (0.63, 1.90)	0.745

**Table 10:** Sociodemographic Correlates of Bullying Perception As a Significant Problem in School: Univariate and Multivariate Logistic Regression Analysis

	N (%)	Unadjusted Odds Ratio (95% CI)	P-value	Adjusted Odds Ratio (95% CI)	P-value
Gender					
Males	338 (57.0)	1.0 (reference)		1.0 (reference)	
Females	225 (43.0)	1.68 (1.18, 2.38)	0.004	1.71 (1.16, 2.52)	**0.006**
Ethnicity					
Arab	353 (60.0)	1.0 (reference)	0.844	1.0 (reference)	
Asian/Caucasian	71 (12.1)	0.96 (0.57, 1.59)	0.860	0.67 (0.38, 1.18)	0.166
Other	164 (27.9)	1.10 (0.75, 1.62)	0.616	0.98 (0.64, 1.50)	0.932
Age Group					
8–10 Years	32 (5.3)	1.0 (reference)	0.420	1.0 (reference)	
11–15 Years	474 (78.3)	0.59 (0.24, 1.42)	0.238	0.858 (0.31, 2.41)	0.772
16–18 Years	99 (16.4)	0.53 (0.20, 1.38)	0.192	0.569 (0.16, 2.06)	0.390
School Grade					
Elementary	129 (21.3)	1.0 (reference)	0.732	1.0 (reference)	
Preparatory	362 (59.8)	0.88 (0.58, 1.34)	0.552	0.90 (0.55, 1.46)	0.663
Secondary	114 (18.8)	1.02 (0.60, 1.73)	0.949	1.30 (0.55, 3.05)	0.551
Years lived in Qatar					
Less than 5 years	74 (12.6)	1.0 (reference)	0.019	1.0 (reference)	
5–10 Years	176 (29.9)	1.99 (1.51, 3.45)	0.014	1.93 (1.06, 3.45)	**0.030**
More than 10 years	338 (57.5)	1.19 (0.75, 1.90)	0.467	1.29 (0.75, 2.20)	0.360

## Discussion

Bullying is a major public health issue that influences the several aspects of health via both short-term and long-term outcomes. There is limited data in literature on the problem of bullying in the Middle East, specifically with regards to GCC countries including Qatar, which makes guided recommendations of several studies inadequately applicable for the aforementioned population. This is the first extensive national cross-sectional study among school students in Qatar investigating bullying and its determinants. Our study showed that school bullying is generally prevalent in Qatar, where students, especially males, have been involved as victims (Table 5), perpetrators (Table 6), both victim and perpetrators (Table 7) but not as observers (Table 9) in all the schools we surveyed. Bala and his colleagues has previously demonstrated a prevalence of 49% of physical bullying in Qatar [17]. The percentage of victims exceeded that of the perpetrators, whereas observers were the most common reporters of bullying, followed by victims. Silva et al. demonstrated similar pattern of reporting. This may reflect students’ willingness to disclose or speak about bullying according to their role within the bullying cycle [18]. Moreover, the predominance of male gender in relation to bullying has been repeatedly highlighted in literature. Khamis, Silva et al., Solberg et al., Scheithauer et al. and Wang et al. demonstrated similar results [18–22]. This has been explained by characteristics of masculinity and social roots that may reinforce aggressive behavior and attitude among males. Nevertheless, Navarro et al. and others studies reported no gender differences in the distribution of school bullying. This may be brought about by the different distribution of masculinity and feminine characteristics among both genders, and by the strategies implemented by each gender to ensure prominence in group and peer relations [18, 23].

Besides gender differences, there is an up-going trend in the frequency of bullying reporting from elementary to preparatory schools, that goes back down in secondary schools. We believe this result is influenced by the students’ tendency to show power in the preparatory level. In contrast, Dake and his colleagues showed that the incidence of bullying in primary school (grades 1–5) is higher than in middle (grades 6–8) and secondary school (grades 9–12), which might also be explained by the biopsychosocial changes associated with puberty and the improved social interaction with age [24].

The most frequent type of bullying reported is verbal bullying as showed in Fig. 1. Silva et al. illustrated a similar result where 1 in 3 students suffered from insults, which could be explained by the underestimation of verbal bullying impacts, which in turn makes it harder to recognize, report and address, compared to other types of bullying [18]. This may partly explain why victims of bullying in our study reported social bullying, which is close to the verbal variant, more frequently compared to other types whereas observers of bullying reported physical bullying as the second most common variant. The sociodemographic correlates of bullying types illustrated in Tables 3, 4 and 5 showed significant variations. A remarkable finding was that preparatory school grade students were more likely to be exposed to all types of bullying compared to other school grades. This same exact pattern has been observed in students in Egypt [25]. These variations can be related to physiological, biological, and psychological changes that accompany different stage of life [26, 27]. On the other side, our results showed that preparators who were males showed more significant interest in practicing of all types of bullying compared to females. Our results redemonstrated similar practices of male perpetrators in Oman [28]. Interestingly, preparators from preparatory school grades practiced more cyber bullying which might be explained by them being introduced to the technologies tools and social media at this particular stage in their education. This pattern continues as students aged 16–18 years had observed more cyber bullying than other age groups.

Interestingly, although most students, especially male subjects, agreed that bullying occurs at school, and acknowledged the significance of the problem, the majority of students reported that they feel safe at school as demonstrated in Tables 9 and 10. This contrasts with other studies in which both victims and perpetrators of bullying were more likely to feel unsafe at school. This result is impressive of the available measures to protect students at school in Qatar, besides being reassuring in terms of the desired students’ perception of the magnitude and impact of the problem. In our study, as seen in Fig. 2, the safest environment reported is the library followed by locker room and computer room, whereas the least safe environment reported is the classroom followed by hallways. In addition, more than half of the students reported sports area as an unsafe place. Comparable results were reported by Vaillancourt and her collaborators. The pattern of safety in the school areas reported may reflect the degree of students’ crowding or the availability of supervising school personnel at the indicated areas [29].

The well-recognized enduring effects of bullying victimization call for an urgent need to prevent and manage the factors as well as the impacts of bullying. The review of Moore et al. reinforced the evidence of a causal association between exposure to bullying and adverse health outcomes including anxiety, depression, poor general health, non-suicidal self-injury, suicidal ideation, and substance use disorders. This can undeniably affect the individual’s biopsychosocial wellbeing in the future and hinder his/her effective contribution to the society which puts him/her at risk of socioeconomic difficulties. They have also shed the light on the dose-response relationship between bullying victimization and suffering from the detrimental effects of concern, which stresses the importance of tackling the repetitive nature of bullying [30].

This study is significant on several grounds. Besides being the first study in Qatar to address the topic of concern, it assessed the phenomenon of bullying from different aspects in terms of frequency, types, determinants, perception in addition to risky environments, aiming to inform future policy-making process and to implement measures that contribute to a healthy school environment, such as education of school personnel and improvement of their readiness to deal with bullying. Moreover, getting insight into students’ knowledge and perception of bullying would provide a chance to design targeted strategies to address their concerns. The study cohort was a relatively large sample from different schools and school types to ensure adequate unbiased selection representation of the population. In addition, the questionnaire used in our study was administered in both Arabic and English, the most common languages used in Qatar, to incorporate the factors related to cultural diversity.

Nevertheless, this study is not without limitations. The cross-sectional study design may not respect the repetitive nature of bullying and it does not allow calculation of incidence. In addition, it precludes making inferences about causality and the direction of influence of both age and gender as significant predictors of bullying involvement. Moreover, the self-reporting-based tool used in the study carries the risk of bias due to potential under-reporting or overreporting. Another important limitation was lack of open questions that could have provided further insight into students’ views. In addition, there were more males than females in the study and only 3 students from grade 12, which underrepresents this cohort. Some important entities were not assessed in the questionnaire, including sexual bullying within types, and bus drop-off area within unsafe environments at school, which are both relevant to frequency and risk of bullying. In addition, the study compared safety of areas at school regardless of different timings, such as break time, before and after classes, free classes and school exit time that may affect risk of bullying at any given area. A significant limitation was taking into account differentiating between students from private and governmental schools. Unfortunately, due to data collection team error, no available data to make distinction between both groups (govermental and private schools) to be able to statistically be analyzed and controlled for. This is considered as an important limitation to this study given the nature of gender segregation policy in governmental schools in Qatar and because it can help, if analyzed, to know the difference in prevalence between the two schools’ system and accordingly plan appropriate interventions. Limitations related to system factors include gender segregation which is applied in governmental schools in contrast to gender-mixed private schools, which may affect frequency and perception of bullying, thus limiting the generalizability of the study findings. Furthermore, students in Qatar are distributed among governmental schools based on their geographical location, meaning that students living in a given geographical location in the country are registered in the same school in that location. It is worth mentioning that generalizability of study results may be limited by the sociodemographic characteristics.

## Conclusion

It is undeniable that achieving the globally targeted goals of Sustainable Development calls for urgent action to tackle the problem of bullying, which is incompatible with healthy growth and sustained well-being. School bullying is a long-standing deep-rooted issue that should never be perceived as a “normal part of growing-up”, regardless of the form it takes, due to its devastating consequences. Generally speaking, the deeply ingrained factors of the bullying epidemic call for a similarly exhaustive multidimensional approaches of management that take in consideration the explicit and concealed driving forces of bullying and attempt to compensate for the pleasure that bullies’ brains are wired to seek out of bullying. We recommend providing adult supervision in all school areas, increasing availability of adults in the areas described as “unsafe” by the students, allocating different break timings for students of different age groups, designing entertaining programs during times of students’ gathering and break times, integrating anti-bullying education within the school curriculum, conducting relevant competitions on anti-bullying knowledge, designing a peer mentor program for perpetrators and conducting future research that takes in consideration the aforementioned limitations of this study with especial focus on comparing governmental to private schools. Schools should always be safe places for children.

### Electronic supplementary material

Below is the link to the electronic supplementary material.

Supplementary Material 1

## Data Availability

The datasets used and/or analyzed during the current study are available from the corresponding author on reasonable request.
