# Supplementary material for: Prevalence and determinants of school bullying in Qatar: a cross-sectional study
Source: BMC Pediatr. 2023 Aug 16;23:400. doi: 10.1186/s12887-023-04227-3 (PMC10428532; doi:10.1186/s12887-023-04227-3)
Supplement: Supplementary file 1 — Supplementary Material 1 [file 12887_2023_4227_MOESM1_ESM.docx]

Questionnaire Supplementary Material given to the students explaining bullying and its types:

**What is Bullying?**

Everyone argues with friends, but bullying is different. Bullying is a relationships problem in which people abuse their power by controlling or harming others who have a hard time defending themselves. Even teasing is bullying when it hurts the other person's feelings or goes on after the person asks for it to stop. It is not bullying if the teasing is done in a friendly or playful way. Also, it's not bullying when people of about equal power argue or fight.

There are lots of different ways to bully someone. Bullying is not an accident. A person who bullies wants to hurt the other person, and does it more than once. Bullying is unfair. The person who bullies has an advantage over the person being victimized. For example who bullies is older, or stronger, or bigger. Sometimes a group of students will gang up on one student.

**Physical bullying examples:**

- Being pushed, hit or kicked.
- Having something thrown at you.
- Having your things taken or broken.

**Verbal bullying examples:**

- Being made fun of by people, being called mean names, or being teased in a mean way.
- Being told that someone is going to harm you.

**Social bullying examples:**

- Having a rumor about you spread.
- People telling others not to be friends with you.
- Having a friendship broken up.
- Being left out on purpose.

**Electronic bullying examples:**

- Having pictures taken of you and posted on the Internet without asking and you don't want them to.
- Having mean instant messages, e-mails, or text messages or pictures sent or
- forwarded about you.
- Having mean messages posted about you on social networking sites such as MySpace and Facebook.
- Having a Web site created that makes fun of you.

**Ethnic bullying examples:**

- Treating you badly because of your skm. co 1o r, re 11· g1· on , or where you come from.
- Telling mean jokes or saying bad things about you because of your skin color, religion, or where you come from.

Disability bullying examples:

- Leaving you out or treating you badly because you can't do something as well as others.
- Making you feel bad because you can't do something as well as others .
- Telling mean jokes about you because you can't do something as well as others.
